# Impact of COVID-19 Mortality on U.S. Life Expectancy by Socioeconomic Rank of County of Residence

**DOI:** 10.1093/geroni/igab046.905

**Published:** 2021-12-17

**Authors:** Denys Dukhovnov, Magali Barbieri

**Affiliations:** University of California, Berkeley, Berkeley, California, United States

## Abstract

Mortality disparities due to COVID-19 pandemic in the US accentuated the gap in the targeted public health and education response among disadvantaged communities. We use county data from John Hopkins University of Medicine in conjunction with county socioeconomic decile rankings, and weekly national data from the Centers for Disease Control to uncover the impact of county-level socioeconomic deprivation on the spatio-temporal dynamic of COVID-19 mortality. We estimate that over the course of 2020, the pandemic reduced the life expectancy at birth by 1.33 years (95% CI 1.0-1.56), and by 0.84 years (95% CI 0.59-1.0) by age 85 across all county SES decile groups, relative to the previous year's projection. The highest losses occurred in counties at the ends of the deprivation spectrum, and least affecting those in its middle. Decomposition of the impact of the COVID-19 mortality by seasonal time periods of 2020 reveals that coastal urban and high-SES counties endured a heavy death toll in the initial stages of the pandemic, though managed to cut it by more than a half by the end of 2020. The least affluent, inland, and rural counties have experienced a dramatic and lasting increase in deaths toward the second half of the year. We find that preexisting socioeconomic disparities before COVID-19 remained in force during the pandemic, leaving populations at all ages residing in underserved communities at a greater risk. This both calls into question and further instructs the ongoing public health interventions enabling more effective and equitable infectious disease mitigation strategies.

